# Rethinking Infrared and Visible Image Fusion from a Heterogeneous Content Synergistic Perception Perspective

**DOI:** 10.3390/s25154658

**Published:** 2025-07-27

**Authors:** Minxian Shen, Gongrui Huang, Mingye Ju, Kai-Kuang Ma

**Affiliations:** 1 School of Internet of Things, Nanjing University of Posts and Telecommunications, Nanjing 210000, China; 1021072323@njupt.edu.cn (M.S.); 1223076715@njupt.edu.cn (G.H.); 2 College of Electronic and Information Engineering, Nanjing University of Aeronautics and Astronautics, Nanjing 210016, China; kkma23@nuaa.edu.cn

**Keywords:** infrared and visible image fusion, generative adversarial network, heterogeneous dual discriminators, information distillation

## Abstract

Infrared and visible image fusion (IVIF) endeavors to amalgamate the thermal radiation characteristics from infrared images with the fine-grained texture details from visible images, aiming to produce fused outputs that are more robust and information-rich. Among the existing methodologies, those based on generative adversarial networks (GANs) have demonstrated considerable promise. However, such approaches are frequently constrained by their reliance on homogeneous discriminators possessing identical architectures, a limitation that can precipitate the emergence of undesirable artifacts in the resultant fused images. To surmount this challenge, this paper introduces HCSPNet, a novel GAN-based framework. HCSPNet distinctively incorporates heterogeneous dual discriminators, meticulously engineered for the fusion of disparate source images inherent in the IVIF task. This architectural design ensures the steadfast preservation of critical information from the source inputs, even when faced with scenarios of image degradation. Specifically, the two structurally distinct discriminators within HCSPNet are augmented with adaptive salient information distillation (ASID) modules, each uniquely structured to align with the intrinsic properties of infrared and visible images. This mechanism impels the discriminators to concentrate on pivotal components during their assessment of whether the fused image has proficiently inherited significant information from the source modalities—namely, the salient thermal signatures from infrared imagery and the detailed textural content from visible imagery—thereby markedly diminishing the occurrence of unwanted artifacts. Comprehensive experimentation conducted across multiple publicly available datasets substantiates the preeminence and generalization capabilities of HCSPNet, underscoring its significant potential for practical deployment. Additionally, we also prove that our proposed heterogeneous dual discriminators can serve as a plug-and-play structure to improve the performance of existing GAN-based methods.

## 1. Introduction

**Figure 1:**
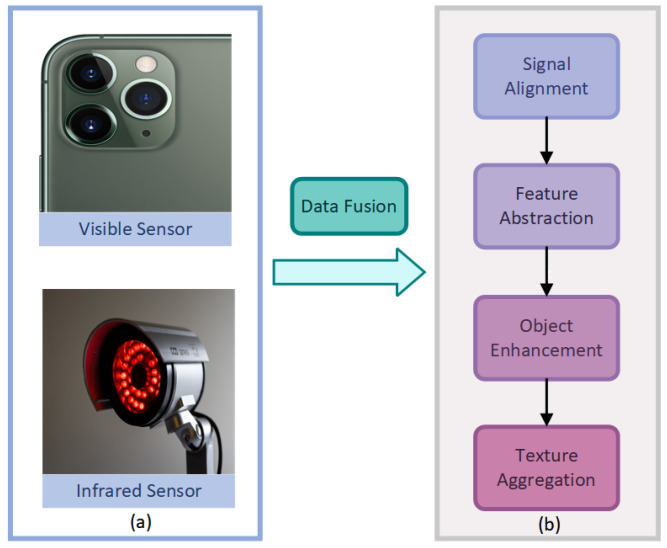
System architecture of the proposed method: (**a**) Integration of smartphone visible sensors with infrared sensors, and (**b**) is the fusion procedure of our method.

The primary objective of infrared and visible image fusion (IVIF) is to synergistically combine the complementary strengths of two distinct imaging modalities, outlined as follows: infrared sensors, which capture thermal radiation to highlight objects, and visible sensors, which provide rich textural details of the scene [1,2,3,4,5]. Effective fusion yields a composite image that is not only more informative but also exhibits enhanced robustness in challenging or degraded environments, such as low-light conditions or scenes obscured by smoke. This improved representation is crucial for a variety of downstream visual tasks, including object detection, surveillance, and autonomous navigation. Indeed, emerging applications, for instance in smartphone photography, stand to benefit significantly from IVIF’s capacity to improve imaging quality and adaptability to diverse environmental conditions, improving real-time fusion on consumer devices equipped with multiple imaging sensors, as depicted in Figure 1.

Current IVIF methodologies can be broadly classified into traditional model-based techniques and contemporary deep learning-based approaches. Traditional methods often depend on handcrafted feature extraction operators (e.g., multi-scale transforms like wavelet or pyramid decompositions and sparse representations) and explicitly defined fusion rules [6]. While these methods can achieve satisfactory results under specific conditions, their inherent limitation lies in their restricted feature representation capabilities, and they often struggle to adapt to the vast diversity of real-world scenes. In contrast, deep learning approaches, particularly those leveraging convolutional neural networks (CNNs) and generative adversarial networks (GANs), have demonstrated superior performance due to their powerful non-linear modeling capabilities, learning intricate fusion strategies directly from data, and producing fused images that are often more visually natural and harmonious [7].

**Figure 2:**
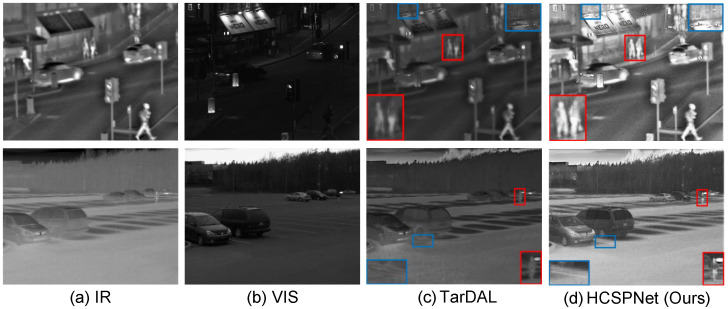
IVIF results under degraded scenarios. The state-of-the-art GAN-based method, TarDAL [8], fails to fully integrate salient information from the source images and often produces undesired artifacts, such as blurring and unnatural texture. In contrast, HCSPNet produces more visually appealing results, owing to its dual heterogeneous discriminators.

Despite these advancements, even state-of-the-art GAN-based IVIF methods can encounter difficulties when confronted with severely degraded input images, such as those where prominent objects are heavily obscured or the entire scene is affected by adverse conditions like dense fog or poor illumination. A common issue in such scenarios is the introduction of undesirable artifacts in the fused output, which can compromise the image quality and the efficacy of subsequent analyses (see Figure 2).

Upon a closer examination of the limitations within existing GAN frameworks for IVIF, a prevalent issue emerges as follows: the predominant use of homogeneous discriminators, i.e., discriminators sharing identical architectural structures. This design choice can inadvertently curtail the discriminative power of the network. If a discriminator cannot accurately differentiate and assess the unique contributions of each source modality within the fused image, the feedback provided to the generator may be suboptimal, potentially leading to the generation of these artifacts. Such a homogenized approach fails to adequately address the intrinsic differences in information content and statistical properties between infrared and visible spectra, thereby imposing a ceiling on achievable fusion performance.

To address these shortcomings, this paper introduces HCSPNet, a pioneering GAN-based framework that, for the first time in the IVIF domain, employs dual discriminators with heterogeneous structures. Our approach re-evaluates the IVIF problem through the lens of “Heterogeneous Content Synergistic Perception”. The central tenet of HCSPNet is to leverage purpose-built, structurally distinct discriminators to more accurately guide the generator in learning to preserve essential information from the source images while concurrently suppressing the formation of artifacts.

The innovative aspects of HCSPNet are manifested in the design of its unique dual-discriminator system. Firstly, each of the two discriminators incorporates a bespoke Adaptive Salient Information Distillation (ASID) module. The internal architecture of these ASID modules is specifically tailored to the distinct characteristics of infrared and visible images, respectively. Their function is to efficiently extract and emphasize the most salient features pertinent to each modality—typically thermal radiation patterns from infrared and intricate textures from visible light. This targeted distillation enables the discriminators to more effectively evaluate the fidelity with which the fused image preserves these critical source components, thereby providing more refined guidance to the generator, while this paper does not focus on changes to the generator’s structure, it highlights how the discriminator system can serve as a plug-and-play enhancement to existing GAN-based methods.

The main contributions of this work are:

- We propose HCSPNet, the pioneering GAN-based framework for IVIF that utilizes heterogeneous dual discriminators. This design is specifically focused on maintaining the integrity of crucial information from source images, especially in challenging, degraded scenarios.
- The two discriminators, with our proposed ASID module, are optimized for their respective sensor modalities, allowing for the simultaneous targeted learning of thermal radiation regions and local details. This framework can serve as a plug-and-play framework for existing GAN-based methods.
- We have conducted extensive experiments on several public IVIF datasets, as well as on related tasks such as medical and biological image fusion. The results not only confirm the superior fusion quality of HCSPNet but also highlight its capability to improve performance in downstream high-level vision tasks and demonstrate the broad applicability of our proposed framework.

The remainder of this paper is organized as follows: Section 2 reviews related work in the field of IVIF. Section 3 details the proposed HCSPNet architecture and its core components. Section 4 presents and analyzes the comprehensive experimental results. Finally, Section 5 concludes the paper.

## 2. Related Works

### 2.1. Model-Based IVIF

Traditional IVIF methodologies focus on designing feature extraction operators and fusion rules to achieve optimal outcomes. These methods can be categorized into four main approaches. The first category is based on the principle that an image can be decomposed into distinct components due to the varying scales of different features. Effective fusion results are achieved by integrating these components using carefully designed rules, such as pyramid transforms [9,10], wavelet transforms [11,12], and curvelet transforms [13,14,15].

Sparse assumption, aiming to enhance the representation of valuable information, learns an over-complete dictionary and characterizes image features using sparse coefficients [16,17,18,19]. Subspace-based fusion methods, recognizing the redundancy inherent in most images, propose mapping images to a low-dimensional subspace to capture essential structural information while effectively reducing interference from redundant data [20,21]. Additionally, hybrid methods, which seek to improve fusion performance by combining the strengths of the aforementioned strategies, have been widely proposed [22,23].

### 2.2. Deep Learning-Based IVIF

Learning-based algorithms leverage the powerful fitting capabilities of neural networks, making them increasingly effective and prominent in the IVIF field. CNN-based frameworks are the most widely used. Liu et al. introduced a multi-focus fusion strategy [24], facilitating fusion tasks by learning the optimal information mapping. Building on this, Li et al. utilized a pre-trained ResNet-50 model to extract deep features from images [25], while Zhang et al. employed sequential convolutional layers to extract features and enhance the fusion results with additional information [26].

Another widely used strategy is based on autoencoders. Li et al. [27] incorporated dense connections in the encoder to enhance information utilization. Furthermore, Li et al. introduced a multi-scale approach that focused on features at various scales to achieve feature reuse [28]. Additionally, Wang et al. combined the strengths of previous models to enable better aggregation of useful features [29].

Despite the generally strong performance, the effectiveness of these methods is constrained by the lack of ground truth data. To address this limitation, Ma et al. introduced the GAN model [30], providing new insights into IVIF. This breakthrough led to the development of several enhanced methods [31,32,33], which will be further discussed in the following subsections.

### 2.3. GANs for IVIF

FusionGAN [30] was the first to introduce GANs into IVIF, providing a novel solution to the challenge of lacking ground truth images in the fusion process. However, FusionGAN primarily evaluates the generated images against visible images, which leads the generator to focus predominantly on the visible image distribution while neglecting the unique characteristics of infrared images.

To address this limitation, Ma et al. proposed DDcGAN [31], incorporating two homogeneous discriminators with identical structures to compel the generator to learn both the detailed features from visible images and the thermal radiation information from infrared images. This dual-discriminator GAN, specifically tailored for image fusion, has inspired the development of several subsequent models. Additionally, GAN-FM [32] enhanced feature learning through improved discriminator architectures, integrating a Markovian discriminator to emphasize local image block structures. Further advancements include TarDAL, presented by Liu et al. [8], which employs a joint training strategy for downstream detection tasks, achieving bi-level optimization between fusion and detection.

In contrast to existing methods, we propose HCSPNet, a pioneering GAN-based framework with heterogeneous discriminators. This novel framework is specifically designed to ensure that fused images effectively retain the essential components from the source images. The two heterogeneous discriminators in this framework serve as a plug-and-play solution for existing GAN-based methods. Extensive experiments demonstrate the superiority of our approach across IVIF, medical image fusion, and biological image fusion tasks, as well as its advancements in downstream high-level vision tasks. Moreover, we showcase the generalization capability of our heterogeneous dual-discriminator framework, which can be seamlessly integrated into existing GAN-based methods in a plug-and-play manner, leading to significant performance improvements.

## 3. Methodology

### 3.1. Overview

**Figure 3:**
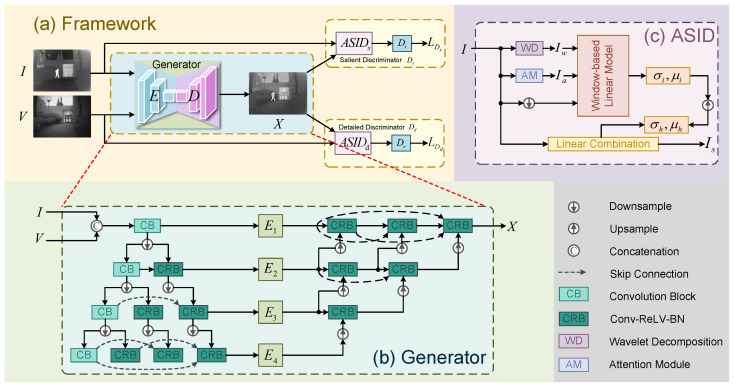
(**a**) Framework of our proposed HCSPNet. (**b**,**c**) Generator and adaptive salient information distillation (ASID) module. Note that we take *I* as the input for an example and the actual input can be *I*, *V*, and *X*.

In this paper, we propose a novel GAN-based framework, termed HCSPNet, which rethinks the IVIF problem from a heterogeneous content synergistic perception perspective. As shown in Figure 3, HCSPNet employs two distinct discriminators, each specifically designed for heterogeneous source images, ensuring that the fused images effectively inherit key components from the source images—namely, the salient thermal radiation information from infrared images and the detailed texture information from visible images.

### 3.2. Generator

Given that this paper focuses on proposing a plug-and-play structure with heterogeneous dual discriminators, we follow the practice of existing methods [27,28,29] to employ a dense connection encoder–decoder structure for image fusion. As depicted in Figure 3, the encoder *E* and decoder *D* of our generator *G* both utilize a multi-scale skip-connected architecture to facilitate information extraction, fusion, and reconstruction, thereby enhancing the robustness of image fusion under degraded conditions. Given an infrared image *I* and a visible image *V* of size W×H, the encoder generates a set of feature maps ${\left\{E_{i}^{k}\right\}}_{k=1}^{4}$ and ${\left\{E_{v}^{k}\right\}}_{k=1}^{4}$ with spatial resolutions of $\frac{H}{2^{k}}\times \frac{W}{2^{k}}$. These extracted features are subsequently passed to the decoder for feature aggregation and reconstruction. To accomplish this, we employ multiple Conv-ReLU-BatchNorm (CRB) modules in conjunction with skip connections. After passing through the final decoder layer, we obtain the fused image *X*, which is then evaluated by the dual heterogeneous discriminators.

### 3.3. Heterogeneous Discriminators

To effectively address the issue of artifact generation and to fully leverage the discriminative capacity, HCSPNet introduces a novel dual-discriminator framework. This framework comprises two structurally distinct discriminators: the salient discriminator $D_{s}$, tailored for assessing infrared-related components, and the detailed discriminator $D_{d}$, designed for visible-light-related components. This heterogeneous design is a cornerstone of the “Heterogeneous Content Synergistic Perception” approach. Specifically, our method incorporates adaptive salient information distillation (ASID) modules, with structures tailored to the unique characteristics of the source inputs. ASID facilitates the extraction of salient and significant information from the fused images, which encourages the discriminators to focus on key components when assessing whether the fused image effectively preserves critical information from the source images.

#### Adaptive Salient Information Distillation Module

To effectively extract salient information from the fused image, we employ the combined guidance of wavelet decomposition and adaptive attention. Given that the fused image must preserve thermal radiation information from the infrared image and detailed texture from the visible image, the low-frequency wavelet coefficients [16] are used to differentiate between the fused image and the infrared image. In contrast, high-frequency wavelets are used to distinguish between the fused and visible images. The adaptive attention mechanism combines spatial attention and channel attention, with their information integrated through a weighted gate mechanism.

As illustrated in Figure 3, we use the infrared image *I* as an example. The Adaptive Salient Information Distillation (ASID) generates the distilled content $I_{s}$, which combines the guidance of the low-frequency information $I_{w}$ and the adaptive attention map $I_{a}$. This approach helps the distilled content emphasize the most salient information. Initially, we generate the down-sampled distilled content $I_{s}^{d}$ using a window-based linear model as follows:

(1) $${\left(I_{s}^{d}\right)}_{i}=\sigma_{w}\mathrm{down}{\left(I\right)}_{i}+\mu_{w},\forall i\in s_{w},$$

where down(·), $s_{w}$, and *i* represent the down-sampling operation, local window, and pixel point *i*, respectively. $\left\{\sigma_{w},\mu_{w}\right\}$ are the linear aggregation coefficients for the pixels in window $s_{w}$, which are optimized by the following objective function $L_{h}$:

(2) $$L_{h}=\sum_{i\in s_{w}}\left[{\left(I_{a}\right)}_{i}^{2}{\left({\left(I_{s}^{d}\right)}_{i}-{\left(I_{w}\right)}_{i}\right)}^{2}+\epsilon \sigma_{w}^{2}\right],$$

where ϵ is a constraint value for $\sigma_{w}$. We define $A_{H}={\left(I_{a}\right)}_{i}{\left(\mathrm{down}\left(I\right)\right)}_{i}$, $A_{L}={\left(I_{a}\right)}_{i}{\left(I_{w}\right)}_{i}$, and $p_{n}$ as the number of the pixels in $s_{w}$. Considering the partial derivatives of the optimization function *L* to $\sigma_{w}$ and $\mu_{w}$ and locating the zero point, we calculate the optimized results for $\left\{\sigma_{w},\mu_{w}\right\}$ as follows:

(3) $$\begin{array}{cc} \; & \frac{\partial L_{h}}{\partial \sigma_{w}}=\sum_{i\in s_{w}}[2\epsilon \sigma_{w}+2\;\mathrm{down}{\left(I\right)}_{i}{\left(I_{a}\right)}_{i}^{2}\\ \; & (\sigma_{w}\mathrm{down}{\left(I\right)}_{i}+\mu_{w}-{\left(I_{w}\right)}_{i})]=0,\\ \; & \Rightarrow \left(\bar{A_{H}^{2}}-p_{n}\times \bar{\bar{{\left(p_{k}^{h}\right)}_{i}}A_{H}}\times \bar{A_{H}}+\epsilon \right)\sigma_{w}=\\ \; & \;\;\left(\bar{A_{H}A_{L}}-p_{n}\times \bar{\bar{{\left(p_{k}^{h}\right)}_{i}}A_{H}}\times \bar{A_{L}}\right),\\ \; & \Rightarrow \sigma_{w}=\frac{\bar{A_{H}A_{L}}-p_{n}\times \bar{\bar{{\left(p_{k}^{h}\right)}_{i}}A_{H}}\times \bar{A_{L}}}{\bar{A_{H}^{2}}-p_{n}\times \bar{\bar{{\left(p_{k}^{h}\right)}_{i}}A_{H}}\times \bar{A_{H}}+\epsilon }, \end{array}$$

(4) $$\begin{array}{cc} \; & \frac{\partial L_{h}}{\partial \mu_{w}}=\sum_{i\in s_{w}}\left[{\left(I_{a}\right)}_{i}^{2}\left(\sigma_{w}\mathrm{down}{\left(I\right)}_{i}+\mu_{w}-{\left(I_{w}\right)}_{i}\right)\right]=0,\\ \; & \Rightarrow \bar{{\left(p_{k}^{h}\right)}_{i}}\;\mu_{w}=\bar{A_{L}}-\sigma_{w}\times \bar{A_{H}},\\ \; & \Rightarrow \mu_{w}=\left(\bar{A_{L}}-\sigma_{w}\times \bar{A_{H}}\right)/\left(\bar{{\left(p_{k}^{h}\right)}_{i}}\right), \end{array}$$

where $\bar{\left(\cdot \right)}$ denote the average operation. For pixels covered by multiple windows, we average those window-wise coefficients $\left\{\sigma_{i},\mu_{i}\right\}$ for pixel *i*. By assembling $\left\{\sigma_{i},\mu_{i}\right\}$ into $\left\{\sigma_{i},\mu_{i}\right\}$, Equation (1) can be rewritten as follows:

(5) $$I_{s}^{d}=\sigma_{i}⊙\mathrm{down}\left(I\right)+\mu_{i},$$

where ⊙ represents the Hadamard product. We then up-sample $\left\{\sigma_{i},\mu_{i}\right\}$ as $\left\{\sigma_{h},\mu_{h}\right\}$ and acquire the high-resolution aggregated feature $f_{k-1}^{h}$ for enriching spatial details as follows:

(6) $$I_{s}={\mathrm{ASID}}_{s}\left(I,I_{w},I_{a}\right)=\sigma_{h}⊙I+\mu_{h}.$$

This process enables the extraction of salient information from visible, infrared, and fused images, encouraging the heterogeneous discriminators to focus on these salient components, thus facilitating the preservation of those important components from the original images and reducing undesired artifacts.

### 3.4. Optimization Strategy

Our HCSPNet is optimized by two parts of the loss functions, i.e., the generator loss ($L_{G}$) and the discriminator loss ($L_{D}$).

(1) *Generator Loss:* Our generator loss consists of the adversarial loss ($L_{\mathrm{ad}}$) and the basic loss ($L_{b}$), generating a high-quality fused image as follows:
  (7) $$L_{G}=L_{\mathrm{ad}}+\alpha L_{b},$$
  where α is the parameter used to balance the two terms. The adversarial loss is defined as
  (8) $$\;L_{\mathrm{ad}}\;=\;E_{X∼d_{X}}\left[{\left(D_{s}\left(X\right)-1\right)}^{2}\right]\;+\;E_{X∼d_{X}}\left[{\left(D_{d}\left(X\right)-1\right)}^{2}\right],$$
  where E means expectation and $d_{X}$ is the distribution of *X*.
  The basic loss $L_{\mathrm{basic}}$ aims to constrain the preservation of important information from source images. Following [7], $L_{\mathrm{basic}}$ is defined as
  (9) $$L_{b}=L_{i}+\beta L_{v},$$
  where
  (10) $$L_{i}={∥X-I∥}_{F}^{2},\;\;L_{v}={∥\nabla \left(X\right)-\nabla \left(V\right)∥}_{1},$$
  where β is the trade-off parameter. ${∥\cdot ∥}_{F}^{2}$ and ${∥\cdot ∥}_{1}$ denote the Frobenius norm and $\ell_{1}$ norm.

(2) *Discriminator Loss:* Discriminator loss $L_{D}$ is combined with the salient discriminator loss $L_{D_{s}}$ and the detailed discriminator loss $L_{D_{d}}$.
  Given that the two heterogeneous discriminators may aggravate the imbalance conflict when the generator learns different features, we jointly train the two discriminators instead of calculating them separately [34,35]. The weight parameter γ is introduced to allow the model to adaptively adjust the optimization to solve the training imbalance arising from the difference of the discriminative structure as follows:
  (11) $$L_{D}=L_{D_{s}}+\gamma L_{D_{d}},$$
  where
  (12) $$\;L_{D_{s}}\;=\;E_{I∼d_{I}}\left[{\left(D_{s}\left(I_{s}\right)-1\right)}^{2}\right]\;+\;E_{X∼d_{X}}\left[{\left(D_{s}\left(X_{s}\right)\right)}^{2}\right],$$
  (13) $$\;L_{D_{d}}\;=\;E_{V∼d_{V}}\left[{\left(D_{d}\left(V_{d}\right)-1\right)}^{2}\right]\;+\;E_{X∼d_{X}}\left[{\left(D_{d}\left(X_{d}\right)\right)}^{2}\right],$$
  where $X_{s}={\mathrm{ASID}}_{s}\left(X\right)$, $X_{d}={\mathrm{ASID}}_{d}\left(X\right)$. $d_{I}$ and $d_{V}$ denote the distributions of *I* and *V*.

## 4. Experiment

### 4.1. Implementation Details

**Datasets:** Our empirical evaluation of HCSPNet was conducted on the following five publicly accessible IVIF datasets: TNO [36], INO [37], RoadScene [38], LLVIP [39], and $M^{3}FD$ [8]. For the TNO dataset (190 images), the latter 100 pairs were used for training and the rest for testing. From RoadScene (221 pairs), the initial 151 pairs formed the training set. For LLVIP (300 pairs selected as per DeRUN [7]) and $M^{3}FD$ (300 pairs), the first 100 pairs from each were allocated for training. To augment the training data, images were uniformly cropped into 128 × 128 pixel blocks, yielding a total of 14,503 training samples.
**Comparative Methods:** We compare our proposed method, HCSPNet, with nine state-of-the-art (SOTA) methods, including U2Fusion [38], FusionGAN [30], DDcGAN [31], GANMcC [40], GAN-FM [32], TarDAL [8], DeRUN [7], GAN-HA [41], DSFD [42], and ReFusion [43]. The selection emphasizes GAN-based techniques to provide a direct comparison for our novel discriminative framework, supplemented by U2Fusion [38] and DeRUN [7] for broader context. All comparisons utilized publicly available code and their originally parameter settings.
**Evaluation Metric:** Following the approaches outlined in [7,17], we utilize six commonly used metrics to evaluate the performance of IVIF methods from multiple perspectives. These metrics include entropy (EN) [44], average gradient (AG) [45], spatial frequency (SF) [46], feature mutual information (FMI) [47], visual information fidelity (VIF) [48], and the universal image quality index (UIQI). EN measures information richness; AG reflects texture detail via gradients; SF indicates the overall activity level and edge information; FMI quantifies shared information between fused and source images; VIF assesses perceptual similarity to source images; and UIQI evaluates structural similarity, luminance, and contrast. Higher values for all these metrics signify better fusion quality.
**Training Details:** The proposed HCSPNet is implemented by PyTorch on two RTX4090 GPUs and is optimized by an Adam optimizer with momentum terms (0.9, 0.999). Our epoch is set as 160 with a batch size of 8. The learning rates are initialized as $2\times 10^{-4}$ and $1\times 10^{-4}$ for the generator and the heterogeneous discriminators, respectively. Furthermore, the hyperparameters α, β, and γ in Equations (7), (9), and (11) are set to 10, 1, and 1, respectively.

### 4.2. Results on the IVIF Task

**Figure 4:**
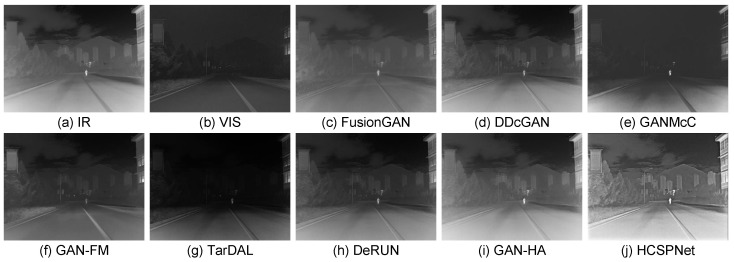
Visual comparisons in low-light scenarios. (**a**,**b**) Infrared image and visible images. (**c**–**j**) Fused images processed by comparison methods.

**Figure 5:**
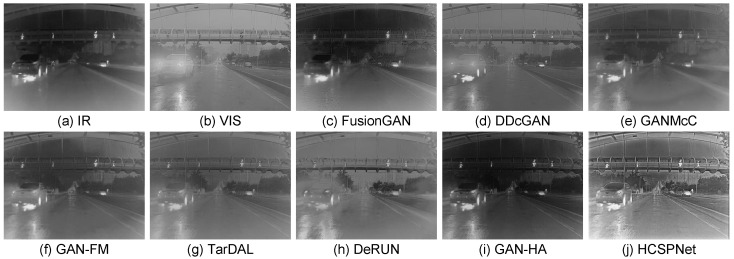
Visual comparisons in glare scenarios. (**a**,**b**) Infrared image and visible images. (**c**–**j**) Fused images processed by comparison methods.

**Quantitative Comparisons:** The average scores for the six evaluation metrics across the five datasets are presented in Table 1. HCSPNet consistently achieved the highest scores for nearly all metrics across all datasets. This demonstrates its superior capability in preserving gradients, textures, enhancing visual quality, and maximizing information content. Notably, HCSPNet often showed substantial improvements over the cutting-edge methods, ReFusion an DSFD, underscoring the efficacy of the heterogeneous content synergistic perception framework.
**Qualitative Comparisons:** To demonstrate the superiority of our model, we qualitatively assess three representative image pairs from the test set and compare the results with the nine state-of-the-art (SOTA) methods. The fusion outcomes of HCSPNet and the comparative algorithms are illustrated in Figure 4 and Figure 5. The evaluation focuses on salient thermal information, represented by bright human body regions, and texture details, such as lane lines, license plates, and bushes.

From a visual perspective, HCSPNet demonstrates several distinct advantages. First, our method closely aligns with the requirements of IVIF by producing fused images that incorporate both infrared and visible information. Notably, HCSPNet preserves a pixel distribution similar to infrared images while maintaining significant texture information from visible images. Second, HCSPNet outperforms other SOTA algorithms in capturing more salient thermal region details and clearer texture information. This can be attributed to the dual discriminator structures designed in our model, which enhance its ability to learn from the source images.

In contrast, competing methods that employ homogeneous discriminators, such as TarDAL, struggle to reconcile information from these disparate sources, often leading to undesirable artifacts. For instance, as shown in Figure 4, TarDAL produces a blurred representation of the human subject under uneven lighting. Furthermore, in Figure 5, it generates unnatural textures on the surface of the moving vehicle, demonstrating a failure to correctly interpret the salient features from the source inputs.

### 4.3. Ablation Study

In this subsection, we conduct ablation studies to validate the efficacy of the HCSPNet structure on the RoadScene dataset, aiming to highlight the practice of our method.

**Effect of our discriminators:** We employ dual heterogeneous discriminators, namely, the salient discriminator ($D_{s}$) and the detailed discriminator ($D_{d}$), to approach the IVIF problem from a heterogeneous content synergistic perception perspective. As demonstrated in Table 2, our method achieves the highest performance when both $D_{s}$ and $D_{d}$ are utilized, underscoring the effectiveness and superiority of our dual-discriminator framework.
**Generalization of our discriminators:** Our dual heterogeneous discriminators, equipped with ASID modules, enhance the extraction of salient information from the fused images, directing the discriminators to concentrate on key components when determining whether the fused images effectively preserve crucial information from the source images. Consequently, our dual-discriminator framework ensures that the generator retains essential content from the source images. In this subsection, we further integrate our dual-discriminator structure into existing GAN-based methods. As shown in Table 3, we observe significant performance improvements when incorporating our framework into other methods, further validating the superiority of our approach.
**Sensitivity analysis of hyperparameters:** In our framework, the final loss function is balanced by three key hyperparameters: α, β, and γ. To analyze their impact on model performance, we conduct a sensitivity analysis by varying each parameter while keeping the others at their default values (α=10, β=1, γ=1). The quantitative results of this analysis on the RoadScene dataset are presented in Table 4. As shown, our chosen set of parameters consistently yields the best or nearly the best results across all six evaluation metrics (EN, AG, SF, FMI, VIF, and UIQI), while performance varies with different hyperparameter values, the model demonstrates robust performance across a reasonable range, indicating that it is not overly sensitive to the precise choice of these parameters. This analysis confirms the rationale behind our selected hyperparameters, which effectively balance the contributions of the adversarial loss, the content loss, and the dual-discriminator losses to achieve optimal fusion quality.

### 4.4. Subsequent Application Verification

In addition to its role as a low-level vision task, IVIF can also function as a pre-processing step that integrates thermal information from infrared images with texture details from visible images. This fusion enhances the performance of high-level visual tasks. To validate this, we apply the proposed model to both object detection and object tracking tasks.

**Figure 6:**
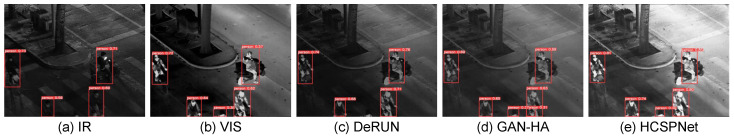
Object detection on fused images, where we select yolov5 as the detector.

**Figure 7:**
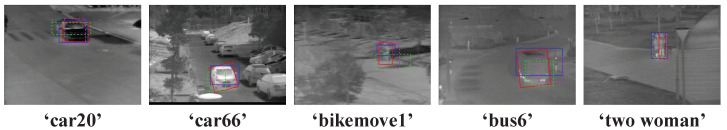
Object tracking on fused images, where we select GFSDCF as the tracker. For easy observation and comparison, we follow the procedure of MDLatLRR [51] and add all tracking notations from different methods into the visible image. In the resulting figures, the bounding boxes are color-coded as follows: the ground truth is denoted by a red box, while the tracking results for DeRUN, GAN-HA, and our proposed HCSPNet are indicated by purple, green, and blue boxes, respectively.

**Object detection:** Object detection is a widely used machine vision technique [49] for identifying specific objects, such as people, vehicles, and other important entities, within images. Fused images with enhanced quality of salient information are expected to yield better downstream performance compared to individual images. Furthermore, algorithms that effectively preserve salient information from heterogeneous source images tend to outperform others in object detection tasks. Following the methodology in [7,50], we employ the general-purpose detector YOLOv5 for object detection experiments on the LLVIP and $M^{3}FD$ datasets. The results for these two datasets are presented in Table 5 and Figure 6. In this context, Precision indicates the probability of correctly detecting true positives among all detected objects, with higher precision implying more accurate identification of real samples. Recall measures the probability of correctly recognizing all positive samples, with higher recall indicating fewer missed targets. The mean average precision (mAP) serves as a comprehensive metric that balances Precision and Recall to assess model performance. The mAP score ranges from 0 to 1, with values closer to 1 representing better performance. The mAP*@*.5 and mAP*@*.95 correspond to the mAP values at confidence thresholds of 0.5 and 0.95, respectively. The results clearly demonstrate that HCSPNet achieves superior performance across these metrics.
**Visual tracking:** Visual tracking, which aims to localize the position of specific objects in video sequences, is a crucial task in computer vision. Following the methodology in [51], we apply our algorithm to the VOT-RGBT sub-challenge, which focuses on short-term tracking as part of VOT2020, to comparatively assess the impact of the fusion approach on tracking performance. The VOT-RGBT benchmark, as outlined in [33,52], consists of 60 video sequences with aligned infrared and visible image pairs. For evaluation, we employ the following two trackers: the learning adaptive discriminative correlation filter (LADCF) [53] and the group feature selection and discriminative filter (GFSDCF) [54]. LADCF demonstrated top performance in the VOT-ST2018 sub-challenge, while GFSDCF further refines LADCF by eliminating redundant information using a group feature selection strategy. Both trackers’ codes are publicly available. For quantitative analysis, we use the following three metrics to evaluate tracking performance: Accuracy, Failure, and Expected Average Overlap (EAO). Accuracy measures the average overlap between the predicted bounding boxes and the ground truth, while Failure assesses tracker robustness. EAO evaluates the expected average overlap between the tracker’s output and the ground truth over time. For both Accuracy and EAO, higher values indicate better performance, while lower Failure values signify better robustness. The results are shown in Table 6 and Figure 7. HCSPNet demonstrates superior performance in enhancing visual tracking compared to other fusion methods.

### 4.5. Extension of the Proposed HCSPNet

We further validate the effect of our methods in the medical image fusion and biological image fusion tasks, which are highly similar to the infrared and visible image fusion task for the heterogeneous sources. Following the common practice, we adopt EN, AG, VIF, and UIQI to evaluate the performance.

**Figure 8:**
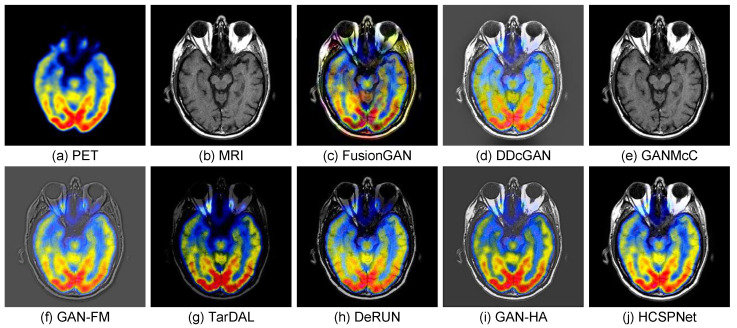
Visual comparisons of medical image fusion. (**a**,**b**) PET and MRI images. (**c**–**j**) Fused images from the compared methods.

**Figure 9:**
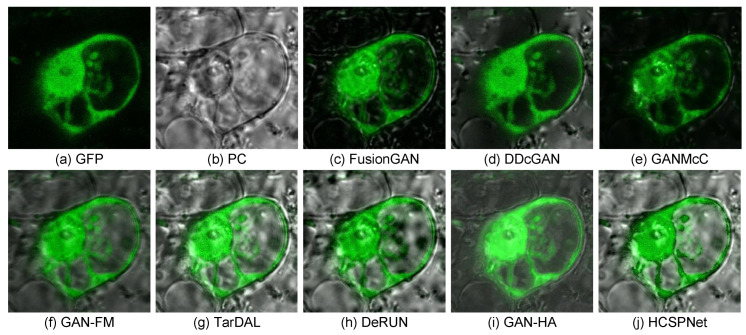
Visual comparisons of biological image fusion. (**a**,**b**) GFP and PC images. (**c**–**j**) Fused images from the compared methods.

(1) *Medical Image Fusion:* For the medical image fusion task, we conduct experiments on PET and MRI image fusion using the HWBA dataset [55], which consists of 144 image pairs for training. We utilize the same evaluation metrics and comparison methods as applied in the IVIF task. The results, as shown in Table 7, indicate that our method achieves superior performance across all metrics, significantly surpassing existing methods. This enhanced performance is primarily due to our proposed heterogeneous content synergistic perception framework and the specifically designed attention module. Visual results of the medical image fusion are presented in Figure 8. These figures illustrate that HCSPNet provides better fusion outcomes with improved salient information compared to other methods, which may suffer from issues related to detail distortion and color component preservation.

(2) *Biological Image Fusion:* In the biological image fusion task, we use the ATC dataset [56] for network training, focusing on the green fluorescent protein (GFP) and phase contrast (PC) image fusion (GFP) task. The training set consists of 60 image pairs. Quantitative results are reported in Table 8. As depicted in Table 8, our method achieves the best performance across all metrics. Additionally, visual comparisons are provided in Figure 9. These figures demonstrate that our approach effectively preserves salient information from GFP and PC images while mitigating phase noise issues from PC images.

## 5. Discussion

Compared to GAN-HA [41], a concurrent work that also addresses the limitations of homogeneous dual discriminators, our method presents clear and significant differences. First, GAN-HA [41] takes a relatively straightforward approach by assuming that crucial information in infrared and visible images is primarily located at the channel and spatial levels. To extract this information, it employs channel attention and spatial attention mechanisms. In contrast, our method introduces an Adaptive Salient Information Distillation (ASID) module, which integrates wavelet decomposition and adaptive attention to guide the extraction of both high-frequency and low-frequency components. This approach enables the heterogeneous discriminators to focus more effectively on salient features, enhancing the preservation of critical details from the original images and further reducing unwanted artifacts. These differences clearly distinguish our approach from that of GAN-HA [41]. Additionally, quantitative analysis confirms that our method consistently outperforms GAN-HA. When we replace our discriminators with those used in GAN-HA, we observe noticeable performance gains. In summary, we propose a novel GAN-based structure with heterogeneous dual discriminators, which can serve as a plug-and-play framework for existing GAN-based methods.

## 6. Future Work

While HCSPNet has demonstrated superior performance on the task of infrared and visible image fusion, its core principle of “Heterogeneous Content Synergistic Perception” holds significant promise for other challenging image fusion tasks. A particularly promising application domain is remote sensing image fusion, specifically the fusion of panchromatic (Pan) and multispectral (MS) images. In this scenario, the panchromatic image provides high-resolution spatial details, while the multispectral image contains rich spectral information, creating a complementary relationship highly analogous to that of visible and infrared images.

Therefore, our proposed heterogeneous dual-discriminator framework could be adapted to specifically evaluate whether the fused image simultaneously preserves the spatial fidelity of the Pan image and the spectral fidelity of the MS image—a critical requirement for tasks such as pansharpening. Furthermore, addressing related challenges within this domain, such as noise, could be integrated into our framework. For instance, techniques explored in pan-denoising research (e.g., PWRCTV [57]) represent a relevant direction for integration. Consequently, extending and applying the HCSPNet framework to the field of remote sensing image fusion represents a compelling avenue for future investigation.

## 7. Conclusions

In this paper, we have introduced HCSPNet, a novel generative adversarial network framework designed to address critical limitations in existing infrared and visible image fusion techniques. Traditional GAN-based methods, often constrained by homogeneous discriminator architectures, are prone to generating artifacts and may struggle in scenarios with degraded image quality. HCSPNet overcomes these issues by pioneering a “Heterogeneous Content Synergistic Perception” approach, which materializes as a dual-discriminator system where each discriminator is structurally distinct and specifically tailored to the unique characteristics of either infrared or visible source imagery. These specialized discriminators are further enhanced by ASID modules, which direct their focus towards the most critical information within each modality—thermal signatures and textural details. The comprehensive experimental evaluations conducted on multiple public IVIF datasets, as well as on analogous medical and biological image fusion tasks, unequivocally demonstrate the superiority of HCSPNet. Furthermore, the application of HCSPNet-fused images has shown tangible benefits in downstream tasks such as object detection and visual tracking, highlighting its practical utility. The successful generalization of its core principles to other fusion domains further underscores the robustness and broad applicability of our proposed framework. HCSPNet, therefore, represents a significant advancement in the field of multi-sensor image fusion, offering a more effective and reliable solution for integrating information from heterogeneous sensor sources.

## Figures and Tables

**Table 1:** Average scores of the six metrics in INO, TNO, RoadScene, LLVIP, and $M^{3}FD$ datasets, with the best result in red. * Indicates the method does not release the official code.

Data	Metrics	U2Fusion	FusionGAN	DDcGAN	GANMcC	GAN-FM	TarDAL	DeRUN	GAN-HA	DSFD *	ReFusion	HCSPNet
TNO	EN ↑	6.612	6.828	6.619	6.758	6.384	6.567	6.841	6.265	—	6.675	6.974
	AG ↑	4.016	3.759	3.953	2.666	2.296	2.493	4.151	4.032	—	4.208	4.369
	SF ↑	10.677	8.888	11.051	10.674	11.874	12.162	12.825	11.767	—	11.186	13.720
	FMI ↑	0.878	0.868	0.875	0.868	0.875	0.882	0.872	0.853	—	0.879	0.895
	VIF ↑	0.571	0.643	0.645	0.446	0.286	0.456	0.495	0.472	—	0.577	0.646
	UIQI ↑	0.771	0.846	0.582	0.426	0.712	0.752	0.713	0.736	—	0.863	0.868
INO	EN ↑	7.096	7.152	7.263	7.035	6.805	6.985	7.203	7.066	—	7.038	7.429
	AG ↑	3.696	3.007	3.637	2.585	2.377	2.370	3.155	3.705	—	3.816	3.882
	SF ↑	10.151	7.429	9.734	6.481	5.970	5.990	7.869	8.855	—	10.758	11.931
	FMI ↑	0.896	0.898	0.896	0.865	0.858	0.892	0.893	0.896	—	0.896	0.899
	VIF ↑	0.465	0.558	0.655	0.464	0.268	0.457	0.461	0.502	—	0.670	0.738
	UIQI ↑	0.766	0.850	0.623	0.520	0.841	0.776	0.654	0.705	—	0.719	0.843
RoadScene	EN ↑	7.192	7.472	7.111	7.048	7.209	7.431	7.293	7.293	—	7.283	7.499
	AG ↑	3.529	5.102	5.002	3.550	3.409	3.818	4.801	5.173	—	5.232	5.263
	SF ↑	9.904	12.883	13.646	8.608	8.833	9.122	12.560	14.035	8.089	14.206	14.067
	FMI ↑	0.858	0.848	0.839	0.847	0.850	0.861	0.854	0.862	—	0.866	0.868
	VIF ↑	0.229	0.442	0.463	0.337	0.274	0.427	0.420	0.453	0.844	0.459	0.465
	UIQI ↑	0.844	0.807	0.699	0.434	0.720	0.731	0.769	0.832	—	0.863	0.868
LLVIP	EN ↑	7.126	6.924	5.263	6.861	6.365	6.708	7.338	7.108	—	7.502	7.695
	AG ↑	3.748	2.903	2.365	2.041	2.044	2.276	3.082	3.352	—	4.315	4.676
	SF ↑	13.737	9.259	10.847	5.728	7.308	7.432	10.348	15.325	—	15.023	15.577
	FMI ↑	0.904	0.902	0.903	0.899	0.901	0.901	0.903	0.900	—	0.906	0.914
	VIF ↑	0.405	0.431	0.283	0.362	0.206	0.364	0.470	1.001	—	0.973	1.053
	UIQI ↑	0.785	0.275	0.436	0.769	0.824	0.533	0.694	0.703	—	0.823	0.848
$M^{3}FD$	EN ↑	6.428	6.728	6.843	6.428	6.639	6.780	7.085	7.536	—	7.712	7.803
	AG ↑	4.239	3.640	4.240	2.411	2.769	2.719	3.671	4.237	—	5.020	5.483
	SF ↑	12.822	10.072	12.881	6.458	8.355	7.654	10.768	12.308	14.764	15.362	16.226
	FMI ↑	0.887	0.881	0.871	0.866	0.878	0.874	0.878	0.883	—	0.888	0.891
	VIF ↑	0.349	0.445	0.603	0.302	0.211	0.381	0.378	0.857	0.844	0.875	0.892
	UIQI ↑	0.721	0.609	0.432	0.806	0.809	0.626	0.814	0.823	—	0.883	0.886

**Table 2:** Ablation studies of our HCSPNet.

Methods	RoadScene					
	**EN ↑**	**AG ↑**	**SF ↑**	**FMI ↑**	**VIF ↑**	**UIQI ↑**
w/o ASID	4.536	3.361	8.024	0.779	0.332	0.833
w/$D_{s}$	5.148	4.904	12.604	0.843	0.368	0.850
w/$D_{d}$	5.137	3.883	10.500	0.847	0.258	0.842
Ours	**7.499**	**5.263**	**14.067**	**0.868**	**0.465**	**0.868**

**Table 3:** Generalization of our dual-discriminators framework, where “+” denotes incorporating our framework into existing GAN-based methods.

Methods	RoadScene					
	**EN ↑**	**AG ↑**	**SF ↑**	**FMI ↑**	**VIF ↑**	**UIQI ↑**
GANMcC	7.048	3.550	8.608	0.847	0.337	0.434
GANMcC+	7.135	3.676	8.623	0.850	0.341	0.439
GAM-FM	7.209	3.409	8.833	0.850	0.274	0.720
GAM-FM+	7.404	3.886	10.017	0.860	0.360	0.735
TarDAL	7.431	3.818	9.122	0.861	0.427	0.731
TarDAL+	7.616	4.253	11.705	0.870	0.467	0.778
GAN-HA	7.293	5.173	14.035	0.862	0.453	0.832
GAN-HA+	7.306	5.186	14.336	0.875	0.463	0.837

**Table 4:** Sensitivity analysis of α, β, and γ.

Metrics	α				β				γ			
	**0.1**	**1**	**10 (Ours)**	**100**	**0.1**	**1 (Ours)**	**10**	**100**	**0.1**	**1 (Ours)**	**10**	**100**
EN ↑	7.069	7.167	7.499	7.273	7.196	7.499	7.203	6.949	7.185	7.499	7.207	6.975
AG ↑	4.415	5.037	5.263	4.386	5.076	5.263	5.217	5.027	4.778	5.263	5.015	4.615
SF ↑	11.326	13.308	14.067	12.219	13.318	14.067	14.003	12.893	13.014	14.067	13.716	13.805
FMI ↑	0.861	0.860	0.868	0.855	0.865	0.868	0.866	0.866	0.863	0.868	0.862	0.860
VIF ↑	0.366	0.427	0.465	0.437	0.453	0.465	0.450	0.438	0.447	0.465	0.452	0.426
UIQI ↑	0.849	0.861	0.868	0.858	0.856	0.868	0.853	0.862	0.857	0.868	0.860	0.852

**Table 5:** Object detection on LLVIP and $M^{3}FD$ datasets with yolov5, where the best result is in red. We only make comparisons with cutting-edge methods.

Datasets	LLVIP				$M^{3}\mathrm{FD}$			
**Metrics**	**Precision ↑**	**Recall ↑**	**mAP*@*.5 ↑**	**mAP*@*.95 ↑**	**Precision ↑**	**Recall ↑**	**mAP*@*.5 ↑**	**mAP*@*.95 ↑**
Infrared	0.929	0.849	0.905	0.472	0.793	0.547	0.603	0.359
Visible	0.931	0.880	0.933	0.521	0.796	0.585	0.645	0.385
GAN-FM	0.928	0.847	0.915	0.467	0.765	0.528	0.579	0.362
TarDAL	0.939	0.882	0.923	0.489	0.766	0.570	0.618	0.355
DeRUN	0.936	0.885	0.937	0.520	0.736	0.585	0.631	0.368
GAN-HA	0.954	0.880	0.935	0.533	0.813	0.592	0.663	0.373
HCSPNet	0.958	0.885	0.947	0.566	0.825	0.603	0.668	0.406

**Table 6:** Object tracking with different tracking methods, where the best result is shown in red. We only make comparisons with cutting-edge methods.

Tracker	LADCF			GFSDCF		
**Metrics**	**Accuracy ↑**	**Failure ↓**	**EAO ↑**	**Accuracy ↑**	**Failure ↓**	**EAO ↑**
Infrared	0.472	73.48	0.209	0.585	45.71	0.239
Visible	0.407	68.23	0.160	0.438	60.05	0.183
GAN-FM	0.519	41.22	0.195	0.586	38.35	0.262
TarDAL	0.469	48.57	0.178	0.493	46.22	0.203
DeRUN	0.417	36.82	0.173	0.457	60.37	0.178
GAN-HA	0.572	38.47	0.226	0.618	31.45	0.228
HCSPNet	0.596	37.32	0.231	0.630	29.43	0.267

**Table 7:** Quantitative results of the medical image fusion task with PET-MRI data, where the best result is shown in red.

Metrics	U2Fusion	FusionGAN	DDcGAN	GANMcC	GAN-FM	TarDAL	DeRUN	GAN-HA	HCSPNet
EN ↑	4.085	4.223	4.275	3.443	4.271	3.109	3.953	4.813	4.935
AG ↑	5.878	5.916	5.935	4.988	5.367	2.972	3.655	5.537	6.083
VIF ↑	0.506	0.457	0.473	0.298	0.383	0.306	0.517	0.553	0.675
UIQI ↑	0.933	0.857	0.773	0.518	0.556	0.483	0.645	0.682	0.933

**Table 8:** Quantitative results of the biological image fusion with the GFP-PC task, where the best result is shown in red.

Metrics	U2Fusion	FusionGAN	DDcGAN	GANMcC	GAN-FM	TarDAL	DeRUN	GAN-HA	HCSPNet
EN ↑	6.572	6.581	6.672	4.864	6.682	4.857	5.687	6.678	6.935
AG ↑	3.769	3.853	4.738	3.652	4.657	5.384	3.768	5.083	5.169
VIF ↑	0.364	0.513	0.625	0.438	0.702	0.656	0.347	0.567	0.772
UIQI ↑	0.769	0.853	0.862	0.418	0.836	0.398	0.716	0.883	0.903

## Data Availability

The original contributions presented in this study are included in the article. Further inquiries can be directed to the corresponding author.
